# Mapping genetic research in non-communicable disease publications in selected Arab countries: first step towards a guided research agenda

**DOI:** 10.1186/s12961-016-0153-9

**Published:** 2016-11-10

**Authors:** Zeina Jamaluddine, Abla Mehio Sibai, Shahd Othman, Soha Yazbek

**Affiliations:** 1 Medical Laboratory Sciences, American University of Beirut, Riad El Solh, P.O. Box 11-0236, 1107 2020 Beirut, Lebanon; 2 Epidemiology and Population Health, American University of Beirut, Beirut, Lebanon

**Keywords:** Arab world, Genetic research, Non-communicable diseases, Scoping review

## Abstract

**Background:**

In the Arab world, intervention and policy response to non-communicable diseases (NCD) has been weak despite extensive epidemiological evidence highlighting the alarmingly increased prevalence of chronic diseases. Generating genetic information is one key component to promote efficient disease management strategies. This study undertook a scoping review to generate the profile of the undertaken research on genetics of NCD publications in selected Arab countries. An analysis of the research produced examined the extent, range, nature, topic and methods of published research. The study aimed at identifying the gaps in genetic NCD research to inform policy action for NCD prevention and control.

**Methods:**

The scoping review was conducted based on the five-stage methodological framework and included countries in Arab region selected to represent various economies and epidemiological transitions.

**Results:**

The search identified 555 articles that focus on genetics-NCD research in the selected Arab countries over the duration of this study (January 2000 to December 2013). The most commonly conducted research was descriptive and clinically focused, rather than etiologically focused. Country-specific carrier and risk screening studies were not among the top research designs. The genetic component of certain highly heritable diseases, as well as diabetes, obesity, hypertension, chronic lung dysfunction and metabolic syndrome were all under investigated.

**Conclusions:**

This scoping review identified gaps for further research in the context of bioinformatics and genome-wide association studies. Genetic research in the Arab region has to be redirected towards NCDs with the highest morbidity, heritability and health burden within each country. A focused research plan to include community genetics is required for its proper integration in the Arab community.

## Background

Non-communicable diseases (NCD), including cardiovascular diseases (CVD), diabetes and several cancers, remain among the leading causes of adult morbidity and mortality worldwide, with a growing economic burden [1]. The alarming rise in NCD burden in both developed and underdeveloped countries has prompted the World Health Assembly 2008 to provide a global strategy on NCD prevention and control [2]. The strategy aimed to generate informed knowledge and transform it into cost-effective actions directly capable of elevating the burden. The approach provided a general review on the current status of NCD knowledge and research gaps, with genetics being an important domain, among other factors. Generating genetic information is a key component in promoting efficient disease management strategies. In fact, investigations targeting chronic disease genetic variants provide better opportunities for screening, diagnosis, and early effective intervention.

On the knowledge production front, advances in the mapping of the human genome (through the Human Genome Project) have greatly contributed to a better understanding of the genetics basis of NCDs. The presence of one or more gene mutations and/or a combination of alleles connotes a genetic predisposition or susceptibility to a disease. Through the lens of genome-wide association studies (GWAS), around 160 loci have been identified and associated with CVD, adding substantial contribution to our understanding of the disease [3]. Research is still ongoing in order to understand the genetic basis of chronic diseases and projects, such as the Encyclopedia of DNA Elements (ENCODE), are currently annotating the noncoding region of the genome to provide new insight in gene regulation [4]. On the clinical front, integration of genetic information has provided ground-breaking application at different stages of disease management, from risk assessment and screening to diagnosis prognosis and treatment. In the case of breast and ovarian cancer, for example, genotyping is used to determine susceptibility to the disease, predict likelihood of recurrence and inform treatment [5]. Furthermore, revelation of novel biological pathways in type 2 diabetes predictions offers hope in providing early intervention, prevention and progression management. The common variant in the gene encoding the transcription factor 7-like gene (*TCF7L2*) confers greater diabetes risk (odds ratio, 1.81) to carriers compared to non-variant carriers [6, 7]. Genome-based tools have been also applied in pharmaceutical clinical practice for proper disease management. Pharmacogenomics has provided an opportunity for optimized drug dosing to avoid toxicity and improve efficacy, such as the case of *CYP2C9*/*VKORC1* and warfarin [8]. In the context of behavioural modification, it has been documented that genetic knowledge plays a role in increased compliance to interventions. Dietary recommendations based on genetics were reported to be more comprehensible and resulted in longer-term weight reduction compared to normal dietary recommendations [9, 10].

Investigating genetics of NCD while taking into consideration the specific populations and ethnic variations offer a specifically efficient disease management. Different ethnic groups have reported differences in gene polymorphism frequencies and have identified new genetic variations. Frequency of leiomyoma-related gene polymorphism differs significantly based on ethnic background; for example, African American women had a higher frequency of the *PP* genotype (35 %) compared to Caucasian (13 %) and Hispanic (16 %) women [11]. Moreover, many novel genetic variations have been noted in different communities. A molecular study in Lebanese adults has identified a differential duplication of an intron region in a *NFATC1* gene associated with congenital heart disease [12].

In the Arab world, policy response to the rise in NCD has been relatively weak despite the extensive epidemiological evidence on its prevalence [13]. To be able to adopt and accommodate global and regional strategies in response to the NCD burden, it is important to identify and analyse the available knowledge, perform regional cross-country comparisons and align national needs with an efficient research agenda. With regards to genetic research in the Arab world, several efforts are worth noting to increase genetic research in the region. In Saudi Arabia, the Saudi Human Genome Program was set up as a research centre to identify the genetic basis of inherited diseases in the Saudi population [14]. In the United Arab Emirates, a biannual conference takes place (Pan Arab Human Genetics Conference) in order to provide a platform for regional and international genetics researchers to meet and discuss genetics-related topics [15]. Data from the ‘Catalogue for Transmission Genetics in Arabs’ database, curated by the Center for Arab Genomic Studies, identified more than 60 recessive genes in the region [16, 17]. Despite those efforts and progress, several papers noted the need for additional comprehensive genetic research targeted at understanding the molecular etiology of genetic related diseases in the Arab world [18].

The study was undertaken to map publications on genetics of NCD from 2000–2013 in selected representative Arab countries. The analysis will generate a profile of the undertaken research both at the level of quantity and quality, highlight disparities between research output and country-specific needs and disease burden, serve as a foundation of systemic review, provide a database of the articles covering this research area and, finally, when disseminated, help redirect research agendas to generate knowledge that would have high impact on disease burden alleviation.

## Methods

### Identification of the relevant studies

The current study stems from a broad scoping review analysis of NCD publications in selected Arab countries following Arksey’s and O’Malley’s five-stages methodological framework: identification of the research purpose, identification of relevant studies, study selection, data charting, and results summary [19]. The countries were selected to represent varied economies and epidemiologic transitions and included Bahrain, Iraq, Kuwait, Lebanon, Morocco, Palestine and Sudan. These countries are classified based on World Bank Gross Domestic Product data into low middle-income countries (Sudan, Palestine and Morocco), upper middle-income countries (Iraq and Lebanon), and high-income countries (Bahrain and Kuwait) [20].

An extensive systematic library search was completed based on probes proposed by a specialized team. The literature search was conducted electronically using PubMed, searching articles from January 1, 2000, until December 31, 2013. The search strategy included, among others, a genetic component using the following terms: genetic polymorphism OR gene polymorphism OR genetic pedigree OR SNP OR GWAS OR genotype OR gene variants OR mutation OR risk allele OR genetic risk factors OR genetic analysis OR candidate gene OR gene. NCD outcomes focused on the four prominent conditions: CVDs, cancers, chronic obstructive pulmonary disease and diabetes. NCD-related terms used for the search strategy included coronary artery disease OR myocardial infarction OR angina pectoris OR peripheral vascular disease OR peripheral arterial disease OR atherosclerosis OR thrombosis OR hypertension OR primary hypertension OR essential hypertension OR secondary hypertension OR stroke OR cerebrovascular accident OR ischemic stroke OR hemorrhagic stroke OR vascular dementia OR transient ischemic attack OR metabolic syndrome OR dyslipidemia OR hyperlipidemia OR combined hyperlipidemia OR hyperlipoproteinemia OR hyperchylomicronemia OR hypertriglyceridemia OR hypercholesterolemia OR familial hypercholesterolemia OR lipoprotein (a) OR type 1 diabetes mellitus OR type 2 diabetes mellitus OR hyperglycemia OR high blood glucose OR glucose impairment OR secondary diabetes OR gestational diabetes OR fasting blood glucose OR hypoglycemia OR hyperinsulinemia OR HBA1c OR diabetic complications OR diabetic nephropathy OR diabetic retinopathy OR diabetic ketoacidosis OR diabetic septic foot OR diabetic neuropathy OR non ketotic diabetic coma OR chronic obstructive pulmonary disease OR chronic bronchitis OR bronchiectasis OR emphysema OR asthma OR lung fibrosis OR pulmonary fibrosis OR lung hyperinflation OR dyspnea OR bronchodilators OR pulmonary function OR cancer OR neoplasm OR malignancy OR tumor OR radiotherapy OR chemotherapy OR biopsy OR tumor markers.

**Fig. 1:**
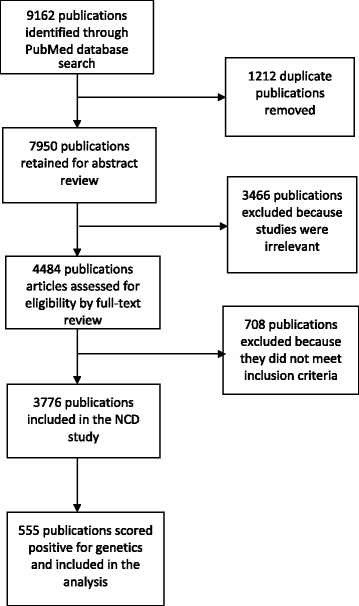
Literature search flow

A total of 3776 NCD-related unique articles were identified, of which 555 articles scored positive if they included a genetic component (Fig. 1). A detailed search strategy is available upon request from the authors.

### Charting the data

A review was conducted on the chosen genetic articles assessing the compatibility and comprehensiveness of the publications included. Data extraction from each of the full-text articles was performed using a detailed data extraction form developed by the researchers. In order to refine the final categories, the form was first piloted on 20 publications before adopting it. The final categories included countries mentioned in the article, impact factor (IF) of the journal, year of publication of article, NCD diseases, NCD subtypes, study population, research design, clinical focus and genetic methodologies. The journals’ IF was reported as per Scimago [21]. Study design was described as review, clinical or experimental. Clinical studies were described as either having a descriptive focus (case report, case series, prevalence studies) or an etiological focus (treatment and causation). The theme of the articles was labelled according to whether the study focused on diagnosis or prevention, control or management, and complications or policy/health system or prevalence. Genetic methodologies included in the article were also reported (proteomics, sequencing, gene expression, pharmacogenomics, cell function, molecular genetics, bioinformatics, epigenetics and GWAS).

### Analysis

We first calculated genetic NCD publications per million population to eliminate the effect of more populous countries. The quality (IF) of the publications was assessed by country and differences in IFs between countries were analysed. A time trend of total genetic NCD publications was evaluated as a percentage of publications published per year. A detailed time trend per country covering 2011–2013 publications was also presented. To assess research gaps, a comparison between the percent disease heritability and percent disease literature coverage was first conducted. Additionally, differences between the top three disease literature coverage of the diseases and top three causes of years of life lost per country were evaluated for each country. Research deficit is highlighted when there was a mismatch between knowledge production and genetic NCD disease burdens.

Descriptive analysis was performed using SPSS version 20.1. *χ* ^2^ test and ANOVA post hoc Tukey test were employed for statistical analysis when appropriate. *P* < 0.05 was considered statistically significant.

## Results and Discussion

### Status of genetic NCD publications

**Fig. 2:**
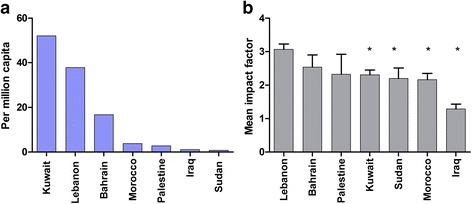
Rates of genetic NCD research and impact factor in selected Arab countries. **a** Studies published per million capita. **b** Mean impact factor. *P < 0.05 was considered as statistically significant when compared to the country with the highest mean impact factor (Lebanon)

The genetic NCD publications represented 15 % of the total NCD publications. Comparative analysis of the 555 publications on the genetics of NCD indicated that it was mostly high- and high-middle-income countries (Kuwait, Lebanon and Bahrain, with the exception of Iraq) which have the highest rate of genetic NCD research per million capita, followed by low-middle- and low-income countries (Morocco, Palestine and Sudan) (Fig. 2a). This finding could be related to the relatively high cost of conducting genetic research, the difficulty of obtaining funds and the stakeholders’ awareness of the value of genetic research. It was noted that, although Kuwait had the highest number of publications per capita, Lebanon published in higher IF journals exceeding those of other countries (Fig. 2b). Notwithstanding the limitations of IF, this may indicate that the knowledge produced on genetic NCD in Lebanon is likely to create a higher impact on the community. High impact research is also capable of generating better funding and has the ability to provide a more perceived and trustful information for evidence-based policy changes [22, 23]. Overall, only 0.4 % of publications were published in very high impact journals (IF above 18), 1.1 % of papers were published in journals with an IF of 18–10, and approximately 6.6 % of the papers were published in journals with an IF of 5–10. The bulk publications (over 73.8 %) appeared in journals with an IF of 1–5 and 18.1 % in journals with an IF below 1.

The top five journals the research was published in included *Anticancer Research*, *PloS One*, *Medical Principles and Practice*, *Leukemia Research*, and *Genetic Testing and Molecular Biomarkers*, hosting around 8.7 % of the total number of publications. Collaboration between multiple Arab countries was noted in only 7.4 % of genetic NCD publications, highlighting the need for more collaborative research among Arab researchers for a better understanding of the etiology of genetic disease in the region. The majority of publications were studies initiated by investigators in the Arab countries and they were carried out at their respective institutions. Although it is interesting to note that 31.7 % of genetic NCD publications were published with western collaborations in western institutions. Pointing out an interest from western communities in genetic diseases in the Arab world. The IF of those publications was not significantly higher than the studies published in the Arab world.

### Time trends in publications of genetic NCD studies

**Fig. 3:**
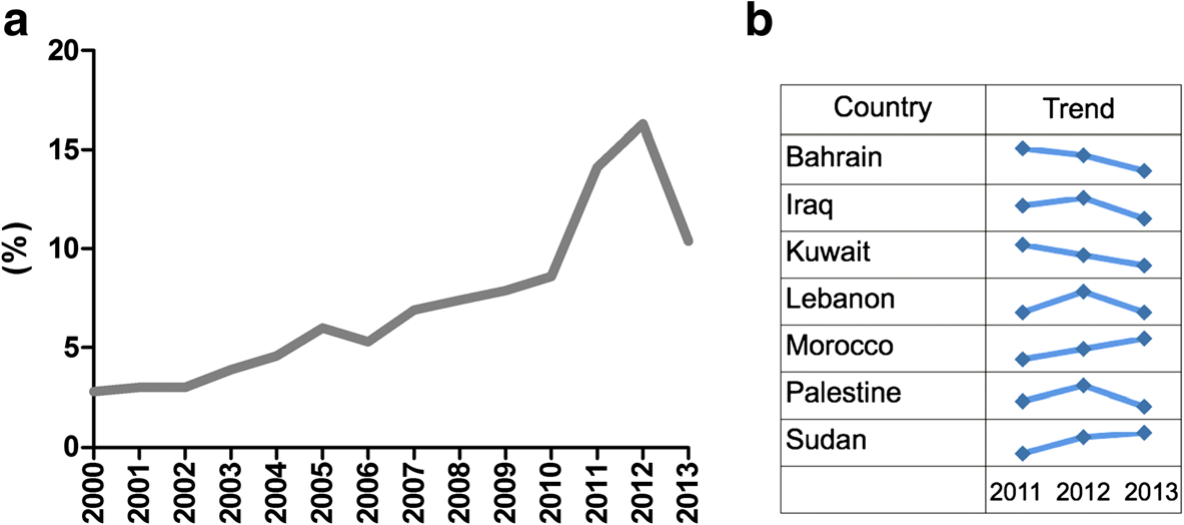
Trends in genetic NCD research publications by time and country. **a** Trend in percent of genetic NCD research published per year from 2010 until 2013. **b** Trends of genetic NCD research publication from 2011 until 2013 by country

Research activity for genetic NCD studies remained low and fluctuating throughout the years until 2010, with a noticeable increase until 2012, and then a drop between 2012 and 2013 (Fig. 3a). When examining the trend in publications per country, we detected that the drop in publications from 2012 until 2013 was mostly attributed to decreased publications in Bahrain, Iraq, Kuwait, Lebanon and Palestine (Fig. 3b). The listed countries have been mostly affected by the recent wars and political and economic turmoil happening in the Arab region. Alternatively, the drop may be attributed to a shift in research funds away from sophisticated costly research, the impact of which will be felt on the long run [24–26].

### Research design and research focus

**Fig. 4:**
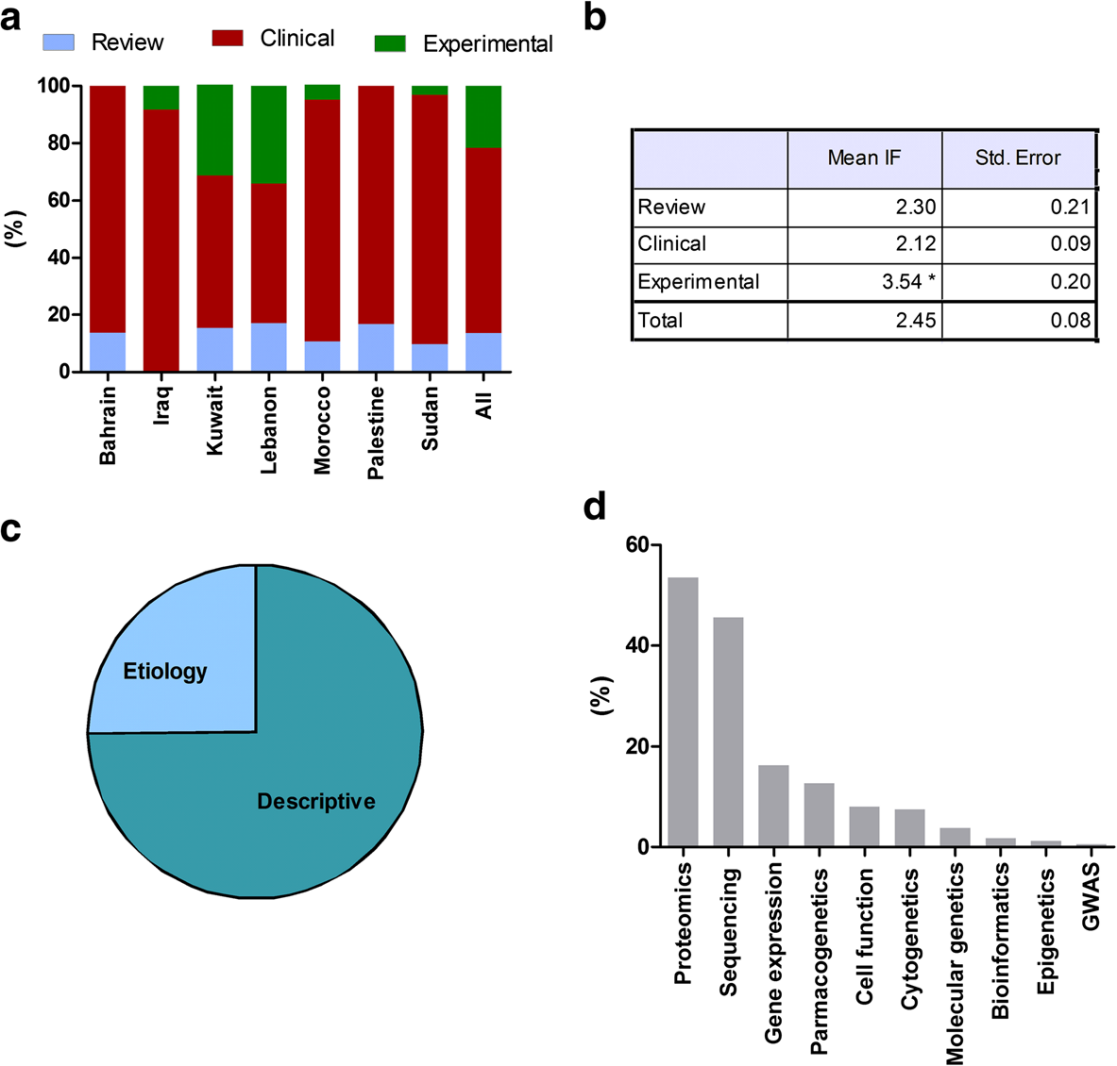
Study type and methods used in genetic NCD research in selected Arab countries. **a** Study designs in total and per country. **b** Mean impact factor by study designs. **c** Descriptive and etiologically focused clinical studies. **d** Genetic methodologies. *P < 0.05 as statistically significant

Clinically focused research constituted the most common type of publications produced across all the countries (64.9 %) (Fig. 4a). This was followed by experimental studies (21.6 %) and reviews (13.5 %). Experimental studies were published in higher impact journals compared to those with a clinical focus or reviews (mean IF 3.540, 2.120 and 2.303, respectively; *P* < 0.05) (Fig. 4b). Among clinical studies, the majority of the published work was descriptive in nature (74.9 %) with a limited focus on etiology (25.1 %) (Fig. 4c). Genetic research methods used in NCD publications focused mainly on sequencing and proteomics with very limited GWAS, epigenetics and cytogenetic studies (Fig. 4d). Although gene-specific sequencing was found to be abundant in the identified papers, the lack of genome-wide sequences for association studies and planned assessment of single nucleotide polymorphisms hinders the ability to develop country-specific programs for genetic risk assessment, information generation and the ability to provide relevant counselling support at the clinical and community level. Furthermore, descriptive studies were more likely to use sequencing than etiological studies (*P* <0.01), while etiologically focused studies have a higher proteomics analysis than descriptive studies (*P* <0.01). Additionally, bioinformatics research was found to be lacking among the identified publications. This deters the ability to include accurate reporting and recording of genetic disorders and genetic profile among medical record systems. The lack of bioinformatics research also highlights the lack of approaches to properly deliver information on genetics and genetic risk factors of NCD to healthcare providers, patients and the community. Limited bioinformatics research may also be related to the lack of expertise in the above discipline and would benefit from directed programs for capacity building in the region.

**Fig. 5:**
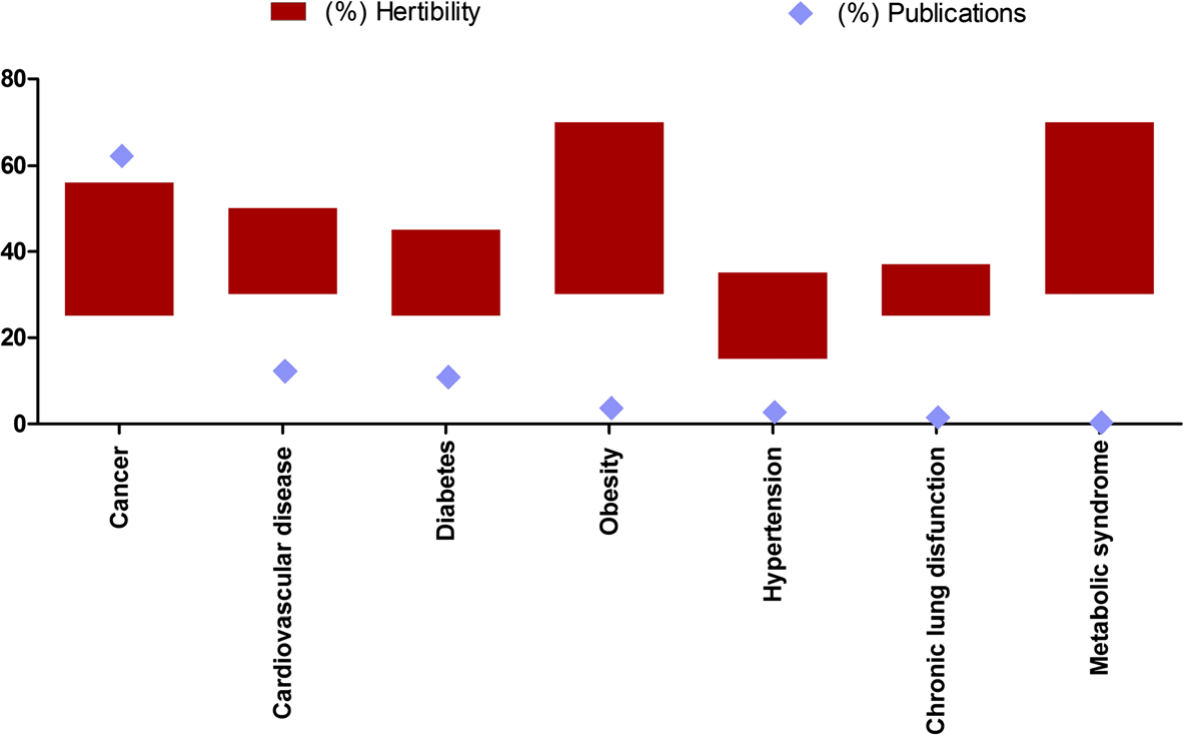
Comparison between percent genetic NCD publications and range percentages of disease heritability

Comparison between percent disease heritability and percentage of publication on specific disease types provided an opportunity to examine the gap between the research focus and the disease priorities (Fig. 4). We identified a surplus of genetic NCD research (62 %) focusing on cancer and that was on account of other highly heritable diseases such as CVD (12.3 %) diabetes (10.9 %), obesity (3.70 %), hypertension (2.80 %), chronic lung dysfunction (1.60 %), metabolic syndrome (0.40 %) and other NCD diseases (6.3 %, renal dysfunction, end stage renal disease, asthma, etc.) (Fig. 5) [27–29]. When examining country-specific publications, the amount of research production and the research focus did not align with country-specific breakdown of NCD burden. If we have a look at the top three researched NCD subtopics in each country and the top three NCD causes of years of life lost in thousands based on the global burden of diseases reports, we notice that the genetic component of most public health priorities topics were under-investigated, including mainly cardiovascular diseases and congenital anomalies, followed by diabetes, all of which are highly heritable disorders [30].

Finally, we denoted a limited focus on community and public health oriented genetic studies. Only 1 % of total genetic NCD studies contributed to the theme of public health policy and community health. WHO research priority clearly stated the importance of developing community genetics services based on genetic approaches in healthcare, yet, our data show limited focus on prevention and population-based genetic studies [2].

## Conclusion

Production of genetic knowledge and informed interventions has shown to reduce the prevalence and severity of NCD [2]. One example of such successful interventions is the ability to reduce risk of cancer by genetic assessment in cases of family cancer syndrome. Genetic testing has proven to play an important critical lifesaving role for family members, such as in the case of colon cancer, for example [31, 32]. Even in countries with very limited resources, simple approaches using genetic knowledge have been successful in single gene disorders and present a potential for success for NCD genetic-based interventions [33, 34]. To adapt global strategies and align our research agendas with evidence-based needs, it is important to generate country- and region-specific information that render the approaches valued in healthcare and effective at the community level.

Findings from the present analysis highlighted several areas of concern. The downward trend in the rate of genetics publications on NCD in countries is worth noting and requires close monitoring over the coming years. The quality of research reporting needs to be improved, judging from the low IF among publications, even in countries with better financial resources. The study also showed that research in the selected countries for the past years has not been aligned with globally identified research priorities in genetics to aid in the prevention and control of NCDs. Country-specific carrier and risk screening is not among the top researched. Thus, identifying country-specific genetic causes and assessment of the magnitude of the burden these genetic risk factors impose on health and the economy will not be possible.

The above findings need to be considered in light of the study limitations. The study is based on selected countries in the region and the search strategy that may have missed reports published in books, articles in the native Arabic language and articles not indexed in PubMed. In spite of this, the study analysis is significant and contributes to the current debate on research value and research waste [35]. Research has to be redirected towards NCD with the highest morbidity, heritability and health burden within each country. Countries listed should develop a research agenda with directed methods to identify the burden of known genetic influenced NCD. Bioinformatics systems should be in place to collect proper genetic information and save genetic and family history. Countries need to develop a plan to introduce community genetics services that are evidence based, highlighting the urgency for research on the topic and proper needs assessment. A focused research plan to include community genetics is required for its proper integration in the Arab community. Producing the proper genetic knowledge would lead to the adaptation of relevant intervention programs, develop techniques to properly implement and evaluate these interventions, and assess their ability to produce favourable outcomes.

## Abbreviations

CVD: Cardiovascular diseases
GWAS: Genome-wide association studies
IF: Impact factor
NCD: Non-communicable diseases
